# Hsp90 inhibition sensitizes DLBCL cells to cisplatin

**DOI:** 10.1007/s00280-022-04407-5

**Published:** 2022-02-21

**Authors:** Linnéa Schmidt, Issa Ismail Issa, Hulda Haraldsdóttir, Jonas Laugård Hald, Alexander Schmitz, Hanne Due, Karen Dybkær

**Affiliations:** 1 Department of Hematology, Aalborg University Hospital, Søndre Skovvej 15, 9000 Aalborg, Denmark; 2 Department of Clinical Medicine, Aalborg University, Aalborg, Denmark; 3 Clinical Cancer Research Center, Aalborg University Hospital, Aalborg, Denmark

**Keywords:** Cisplatin, 17AAG, Hsp90, Drug combination, Diffuse large B-cell lymphoma, DNA repair

## Abstract

**Purpose:**

Platinum-containing therapy is standard treatment for relapsed Diffuse Large B-Cell Lymphoma (DLBCL). However, the efficacy of treatment is limited by drug resistance leading to relapse. Cisplatin resistance has been linked to impairments of the DNA damage response, and several DNA repair proteins have been identified as clients of the molecular chaperone Hsp90. Here, we investigated the combinatory treatment of cisplatin and the Hsp90 inhibitor, 17AAG, in DLBCL cells to evaluate if inhibition of Hsp90 could sensitize DLBCL cells to cisplatin treatment.

**Methods:**

Cell viability was assessed for cisplatin and 17AAG as monotherapies and for 25 different combinations in 7 DLBCL cell lines, where the Bliss Independence Model and the Combination Index were applied to assess their interaction. Induction of apoptosis and DNA damage response were evaluated by measuring Annexin V and γH2AX levels after 48 h of exposure.

**Results:**

17AAG synergized with cisplatin in DLBCL cells as detected in both interaction assessment models, resulting in a lower viability after 48 h for the combination-treated cells compared to both vehicle and single drug-treated cells. The combination also induced a stronger apoptotic response and an increase in DNA damage in 17AAG, cisplatin- and combination-treated cells compared to vehicle-treated cells, with the effect of the combination generally being higher than compared to both single drugs.

**Conclusion:**

This study demonstrates that 17AAG sensitizes DLBCL cells to cisplatin treatment. This effect is correlated with increased apoptotic and DNA damage response, potentially mediated by downregulation of Hsp90 clients in DNA repair pathways. Thus, cisplatin resistance could plausibly be overcome by combining the treatment with an Hsp90 inhibiting drug.

**Supplementary Information:**

The online version contains supplementary material available at 10.1007/s00280-022-04407-5.

## Introduction

Diffuse Large B-Cell Lymphoma (DLBCL) accounts for 30–40% of all adult non-Hodgkin lymphomas [1]. The standard of care treatment includes rituximab, cyclophosphamide, doxorubicin, vincristine, and prednisone (R-CHOP) [2]. Although many patients achieve a long-term remission using this first-line treatment regimen, one third are refractory or relapse after initial remission [3–5]. The treatment regimens most commonly used for refractory and relapsed DLBCL patients (rrDLBCLs) are R-DHAP (rituximab–dexamethasone–cisplatin–cytarabine), R-ICE (rituximab–ifosfamide–carboplatin–etoposide), and R-GDP (rituximab–gemcitabine–dexamethasone–cisplatin), all of which include platinum-based chemotherapeutic drugs [6].

Cisplatin, together with other platinum-based drugs, crosslinks DNA purine bases causing DNA damage and subsequent apoptosis [7]. The Food and Drug Administration (FDA) initially approved cisplatin for treating testicular cancer, and successful anti-neoplastic effects made the drug applicable for treatment of various cancer types, including ovarian, lung and bladder cancer [8], and the drug is currently in use for treatment of relapsed DLBCL [6]. As a mechanism of action, cisplatin induces DNA adducts, e.g., intrastrand adducts and DNA interstrand crosslinks, which block transcription and DNA synthesis. DNA adducts trigger a cellular response to eliminate the lesions, often by cell cycle arrest to allow time for DNA repair and avoidance of inadvertent replication of damaged DNA. DNA repair has been investigated as an intrinsic mechanism for the development of resistance to cisplatin [8]. In addition to a role in cisplatin response, DNA repair mechanisms are essential for development of B cells, contributing to the antibody diversification processes [9], where differentiation-dependent DNA rearrangement in immunoglobulin genes occur. In cancer cells DNA repair is crucial to counteract DNA damage induced by chemotherapeutic drugs, such as cisplatin [10]. The significant challenge of cisplatin treatment is the development of resistance, where the DNA repair machinery has been suggested as a target to overcome resistance [8].

One important player in regulating DNA repair proteins is the chaperone Hsp90, which has many clients involved in DNA repair [11]. The Hsp90 machinery regulates many different processes in the cell, e.g., stabilizing folding intermediates that allow clients to interact with their partners or regulating ubiquitin-mediated proteasome degradation. Under physical conditions, Hsp90 represents around 1–2% of the total cellular protein content, with a pivotal role to buffer proteostasis against environmental stress. Under drug treatment, such as cisplatin treatment, an extreme environmental stress is induced and the Hsp90 reservoir can be exhausted affecting e.g., disease onset such as cancer [11]. Hence, Hsp90 is considered a crucial facilitator of oncoproteins, and is expressed at two–tenfold higher levels in tumor cells than normal cells, and thereby represents a valid anti-cancer drug target [12].

Pre-clinical studies have validated the potential of Hsp90 inhibition in, e.g., suppressing tumor growth and metastatic potential as well as sensitizing tumors to the effect of chemotherapeutic therapies, the latter possibly as a consequence of the inhibition of double strand break (DSB) repair due to the many DNA repair proteins present as Hsp90 clients. Inhibition of Hsp90 has therefore been investigated as an add-on treatment in several cancer types [11]; however, no study has to our knowledge explored its role in cisplatin response in DLBCL.

One well-studied Hsp90 inhibitor is 17-(Allylamino)-17-demethoxygeldanamycin or tanespimycin (17AAG), which binds to Hsp90 and inhibits chaperoning of Hsp90 clients. Many of these clients are involved in DNA repair [11, 13], making Hsp90 an interesting treatment target.

In this study, we wanted to evaluate the effect of combining cisplatin with the Hsp90 inhibitor 17AAG in DLBCL. Our results document a strong synergistic drug interaction in DLBCL cell lines, confirmed using two independent combination interaction measurements. The drug combination was also validated to induce a stronger apoptotic response and an increased DNA damage response compared to single drug treatment.

## Materials and methods

### Cell lines

The human DLBCL cell lines DB, NU-DHL-1, SU-DHL-4 and SU-DHL-5 were obtained from the German Collection of Microorganisms and Cell Cultures GmbH (DSMZ), and HBL-1, OCI-Ly7 and RIVA were kindly provided by Professor Jose A. Martinez-Climent, University of Navarra, Pamplona, Spain. Cells were cultured at 37° C in a humidified atmosphere containing 5% CO_2_ in RPMI-1640 complete medium supplemented with 10% Fetal Bovine Serum (FBS) (20% for SU-DHL-5 and DB) and 1% Penicillin–Streptomycin. All cell lines were authenticated by DNA barcoding [14] and examined for mycoplasma infection throughout the study.

### Drugs, drug screening and drug interaction calculations

Cisplatin was obtained from Aalborg University Hospital and supplied as a 1 mg/mL solution in isotonic water. 17-(Allylamino)-17-demethoxygeldanamycin (17AAG) was purchased from Sigma Aldrich (Cat# A8476), supplied as 500 µg lyophilized powder, dissolved in DMSO and aliquoted in 250 µg/mL. DMSO was used as a vehicle in all wells treated with 17AAG. Dilution series were prepared prior to each experiment, where cisplatin was diluted in isotonic water, and 17AAG was diluted in PBS.

For all dose–response and drug combination experiments, cells were seeded 24 h prior to treatment at cell line-specific concentrations (0.15–0.6 10^6^ cells/mL, Supplementary Table 1) in 96-well tissue culture plates (Cat#655 180, Greiner CELLSTAR), to correct for previously identified differences in cell line growth rates, and exposed for treatment for 48 h before analysis [14, 15].

To assess cell viability, the MTS assay was used (Cat#G3581, CellTiter 96® AQ_ueous_ One Solution Cell Proliferation Assay, Promega, USA) according to manufacturer’s protocol, and the absorbance was measured at 490 nm using a FLUOstar OPTIMA (BMG Labtech, Germany). Each cell line was subjected to 5 concentrations of cisplatin and 17AAG in 4 replicates per concentration, respectively, which were set as twofold increments and decrements from IC50 values determined from pre-set-up estimations for 3 of the 7 cell lines (data not shown). These doses led to 25 combinations for cisplatin + 17AAG combination drug screens in each cell lines (overview in Supplementary Fig. 1 and 2).

To calculate drug interactions, we used the Bliss Independence Model [16, 17], resulting in a Bliss score, or epistasis ($\varepsilon$). The Bliss score ($\varepsilon$) is calculated by comparing the observed fitness of a given system (${W}_{AB}$) to the theoretical fitness (${W}_{A}{W}_{B}$) [17]:

$$\varepsilon ={W}_{AB}-{W}_{A}{W}_{B}.$$

To increase the accuracy of estimating an interaction, the Chou–Talalay method was also applied on all combination experiments using the CompuSyn software (www.combosyn.com) [18–20].

### Annexin V staining for assessment of apoptosis

To detect and analyze apoptosis in DLBCL cells after treatment with cisplatin and/or 17AAG, we performed staining with FITC Annexin V (Biolegend, Cat#640,906). Annexin V binds to phosphatidylserine on the surface of apoptotic cells, which is normally found intracellularly but during early apoptosis it translocates to the external surface of the cell. The analysis makes it possible to identify apoptotic cells. Cells were seeded at 0.5 × 10^6^ cell/mL in triplicates in 12-well plates (Cat#665 180, Greiner CELLSTAR), left for 24 h prior to drug exposure for 48 h (0.415 µg/mL Cisplatin (all cell lines) and 0.68 µg/mL (OCI-Ly7 and RIVA) and 0.17 µg/mL (DB) 17AAG as single doses and in combination). Cells were washed in PBS directly after treatment, stained in Annexin V binding buffer (Annexin V 1:20) and analyzed on a BD FACSCanto II flow cytometer. To set gates between viable and apoptotic cells, positive apoptosis control samples were prepared for each cell line using a 15-min incubation at 60 °C, which were run with and without Annexin V stain.

### Anti-phospho-γH2AX staining

To detect and analyze DNA damage in DLBCL cells treated with cisplatin and/or 17AAG, the FITC anti-H2A.X Phospho (ser139) (phospho-*γ*H2AX) antibody (Biolegend, Cat#613,404) was used. The antibody binds the phosphorylated form of H2A histone family member X (γ*H2AX*) which forms when double-stranded DNA (dsDNA) breaks appear [21]. Cells were exposed to cisplatin and 17AAG in the same manner as described in the previous experiments. After treatment, cells were fixed in 70% ethanol, stored in − 20 °C until analysis and processed according to the protocol supplied by the manufacturer of the antibody, including an optimized dilution of the antibody at 1:40*.* Samples were analyzed on an BD FACSCanto II flow cytometer.

#### Statistical analysis

The program FlowJo v.10.7.1 was used to analyze data from the BD FACSCanto II flow cytometer. For each experiment, data acquisition with specific gating strategies was used to only include the appropriate cells and leave out unwanted particles, such as debris and doublets. GraphPad PRISM 9.0 was used to visualize data and perform statistical analysis. A Student’s Independent *t*-test was used to evaluate *p*-values between single drug- and combination treatment (two groups compared per analysis). Microarray data were Log_2_ normalized and mRNA expressions of *Hsp90AA1*, *Hsp90AB1*, and *Hsp90B1* were extracted in Partek Flow. Correlation between mRNA expression and 17AAG sensitivity was analyzed by Pearson correlation. CEL files are deposited with GEO accession GSE53798 [14].

## Results

### Cisplatin and 17AAG suppress viability in DLBCL cell lines

**Fig. 1:**
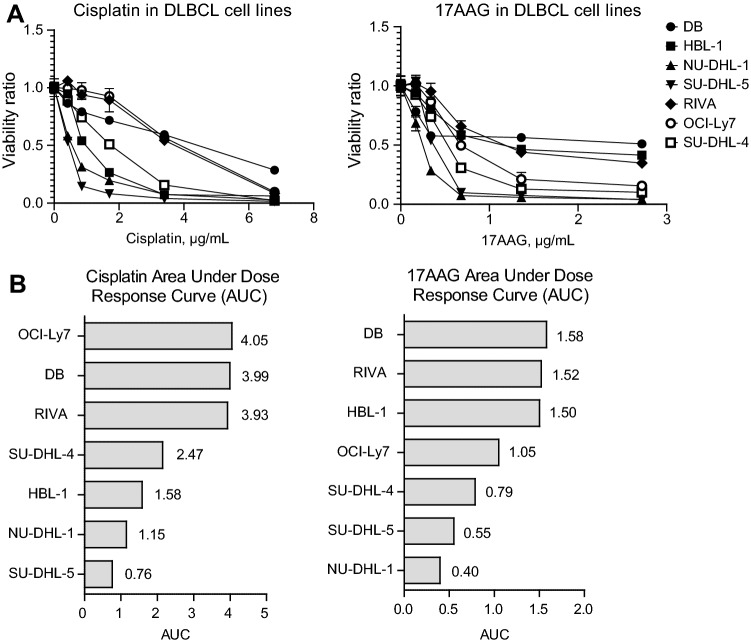
Single drug screening in 7 DLBCL cell lines. **A** Dose–response curves for cisplatin and 17AAG in the 7 DLBCL cell lines DB, HBL-1, NU-DHL-1, SU-DHL-5, RIVA, OCI-Ly7 and SU-DHL-4. Response was calculated as a viability ratio to vehicle-treated controls, where controls are set to 1. **B** Overview of Area Under Dose–Response Curve (AUC) across the 7 cell lines. Cell lines sorted top to bottom from highest AUC, corresponding to lowest response, to lowest AUC, corresponding to highest response

To evaluate the effect of cisplatin and 17AAG in DLBCL, each drug was tested in 5-point dose–response curves in 7 DLBCL cell lines (Fig. 1A). Drug effect was summarized by the Area Under the dose–response Curve (AUC), making results less dependent on variations in the dose–response curves than when using IC50. In agreement with others [22] inhibition of Hsp90 decreased cell viability in DLBCL cells (Fig. 1A). Ranking of cell lines according to AUC showed DB, RIVA and HBL-1 to be the least 17AAG sensitive cells (Fig. 1B), with no observable correlation between 17AAG response and Hsp90 mRNA expression (Supplementary Fig. 3). The cell lines OCI-Ly7, DB and RIVA displayed the lowest sensitivity to cisplatin (Fig. 1B) and was, therefore, of specific interest in further analyses.

### 17AAG enhances cisplatin sensitivity in DLBCL cell lines

To investigate the role of Hsp90 in cisplatin response in DLBCL, 17AAG was tested in combination experiments with cisplatin in the panel of 7 DLBCL cell lines. To facilitate systematic interpretation, comparisons between cell lines, and to incorporate as much as possible of the drug interaction landscape, 5 drug concentrations spanning previously identified IC50 values for all cell lines were selected (Supplementary Table 2). The drug interactions were initially explored using the Bliss Independence Model [16], where the observed response (*W*_AB_) is compared to the theoretical response using single dose responses from participating drugs (*W*_A_ + *W*_B_) resulting in a Bliss score ($\varepsilon$) for each combination. A negative Bliss score ($\varepsilon <0)$ indicates a synergy, a value close to zero ($\varepsilon =0$) indicates an additive effect and a positive value ($\varepsilon >0$) indicates antagonism.

**Fig. 2:**
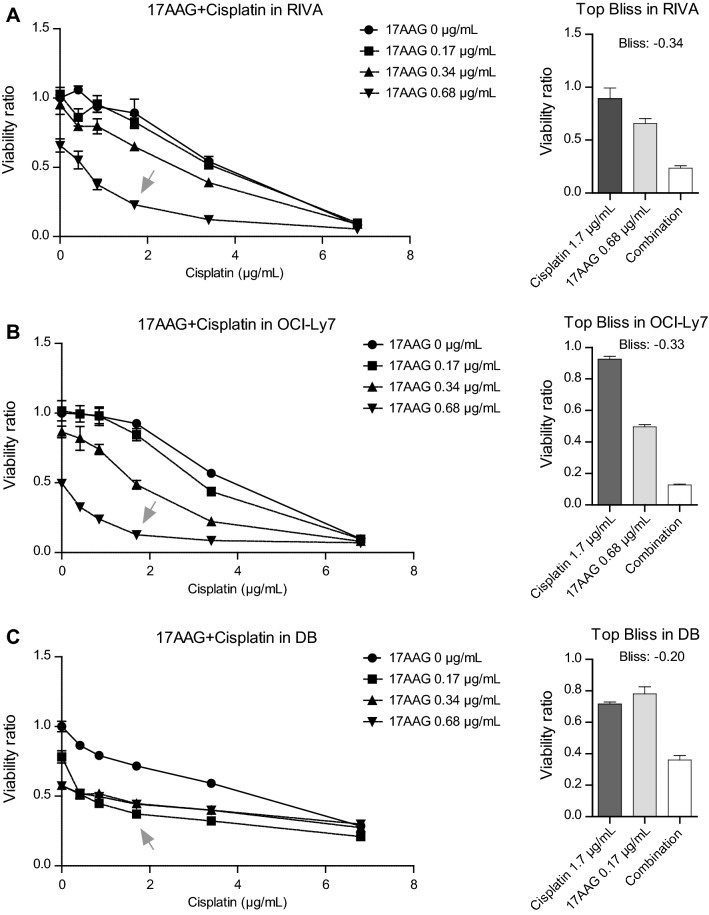
Drug combination screening in DLBCL cell lines. The drug combination cisplatin + 17AAG was tested in a combination matrix consisting of 5 doses of each drug, resulting in a total of 25 different combinations. **A** *Left panel*, dose–response curve for cisplatin at different set doses of 17AAG in RIVA. *Right panel*, combination response compared to both single drugs for the concentration resulting in the highest Bliss score (also marked with a gray arrow in the left panel). **B** *Left panel*, dose–response curve for cisplatin at different set doses of 17AAG in OCI-Ly7. *Right panel*, combination response compared to both single drugs for the concentration resulting in the highest Bliss score (also marked with a gray arrow in the left panel). **C** *Left panel*, dose–response curve for cisplatin at different set doses of 17AAG in DB. *Right panel*, combination response compared to both single drugs for the concentration resulting in the highest Bliss score (also marked with a gray arrow in the left panel). For all graphs, response was calculated as a viability ratio to vehicle-treated controls, where controls are set to 1

**Fig. 3:**
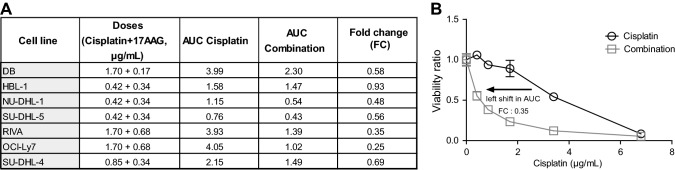
Comparisons of shift in AUC between cisplatin and combination. **A** Table with all 7 DLBCL cell lines showing doses for the combination resulting in the highest Bliss score, AUC for cisplatin and combination and the corresponding fold change, i.e., AUC_combination_/AUC_cisplatin_. **A** low fold change equals a stronger response. **B** Visualization of AUC shift using results for RIVA

Using a 5 × 5 matrix drug combination set-up protocol, we simultaneously recorded the combination effects of a total of 25 combinations (35 data points for each drug combination including single doses), resulting in one drug interaction landscape per drug combination and cell line (Supplementary Figs. 1 and 2). From each drug interaction landscape, the most synergistic doses were selected and are shown as dose–response curves in Fig. 2A-C (gray arrows). The lowest Bliss score value for each cell line ranged between an additive score of − 0,04 in NU-DHL-1 to a strong synergy score of − 0,35 in RIVA (Table 1). The strongest synergy was observed in the least cisplatin sensitive cell lines RIVA, OCI-Ly7 and DB (Fig. 2A-C. Comparison of AUC values from single- and combinatory drug screens in RIVA, OCI-Ly7 and DB revealed a clear decrease in AUC using the combination, demonstrating increased sensitivity (Fig. 3A, exemplified by RIVA in Fig. 3B). In summary, using the Bliss Independence Model, we observed a synergistic effect in 6 of 7 DLBCL cell lines treated with cisplatin in combination with 17AAG. The synergistic effect was particularly strong in the three cell lines RIVA, OCI-Ly7 and DB.

**Table 1:** Summary of strongest Bliss scores across 7 DLBCL cell lines

	Dose for Cisplatin (µg/mL)	Dose for 17AAG (µg/mL)	Strongest Bliss score across 7 DLBCL cell lines for Cisplatin + 17AAG
NU-DHL-1	0.42	0.34	− 0.04
SU-DHL-4	0.85	0.34	− 0.10
SU-DHL-5	0.42	0.34	− 0.14
HBL-1	0.42	0.34	− 0.15
DB	1.70	0.17	− 0.20
OCI-Ly7	1.70	0.68	− 0.33
RIVA	1.70	0.68	− 0.34

**Fig. 4:**
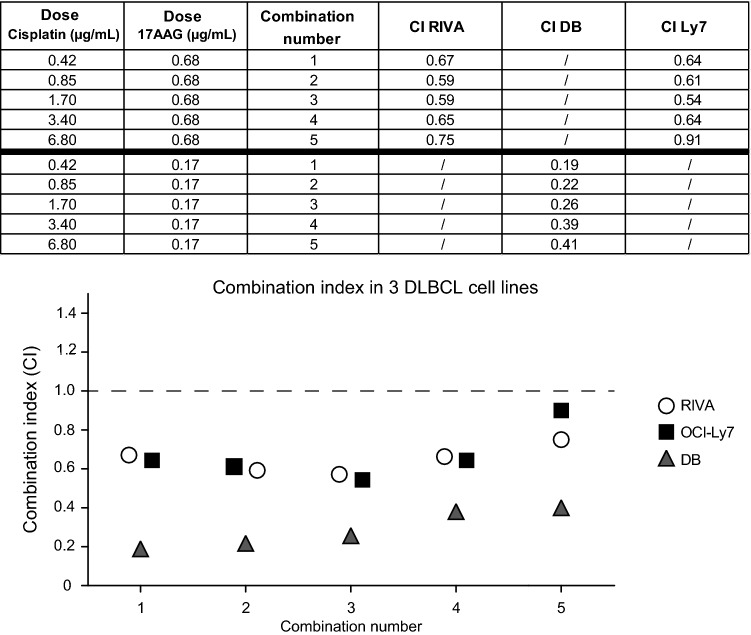
Combination index for combination in DLBCL cell lines. A combination index, an alternative estimation of combination effect, was calculated from the drug combination screen. On the top, a table with Combination index (CI) for RIVA, OCI-Ly7 and DB can be seen. The three columns on the left show the single doses in five different combinations, and the three columns to the right show the CI for each cell line. Visualization of the CI in the three DLBCL cell lines is shown on the bottom. A CI > 1 is an antagonistic interaction, a CI = 0 equals no, or additive, drug interaction, and a CI < 1 equals a synergistic drug interaction

To strengthen our drug interaction results, and to encompass the entire interaction landscape when assessing a combination, we calculated a Combination Index (CI) for each combination using the CompuSyn software. Here, a CI below 1 indicates a synergy, a CI above 1 indicates an antagonistic interaction and a CI equal to 1 indicated no interaction, or an additive interaction. The synergistic interaction was validated in 6 of the 7 cell lines using the CI measurement (Fig. 4 and Supplementary Fig. 4), with RIVA, OCI-Ly7 and DB displaying the strongest synergy (Fig. 4), as observed when applying the Bliss Independence Model (Fig. 2).

### Cisplatin + 17AAG combination induces apoptosis in DLBCL cells

**Fig. 5:**
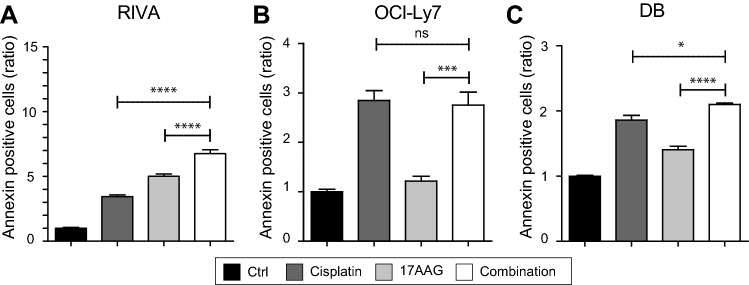
Single drug and combination effect on apoptosis in DLBCL cell lines. Annexin V expression was used to assess the apoptotic response in RIVA, OCI-Ly7 and DB after treatment with single drugs and drug combination for 48 h. **A** Annexin V expression after flow analysis in RIVA. Doses used were 1.7 µg/mL for cisplatin and 0.68 µg/mL for 17AAG. **B** Annexin expression after flow analysis in OCI-Ly7. Doses used were 1.7 µg/mL for cisplatin and 0.68 µg/mL for 17AAG. **C** Annexin expression after flow analysis in DB. Doses used were 1.7 µg/mL for cisplatin and 0.17 µg/mL for 17AAG. All treated cells had values significantly higher than the control cells, except for the 17AAG-treated OCI-Ly7 cells. Response was calculated as a Annexin V expression ratio to vehicle-treated controls, where controls are set to 1. The percentage Annexin V-positive cells were identified using gates from positive controls for each cell line. Student’s Independent *t*-test was used for statistical analysis, *p value < 0.05, **p value < 0.01, ***p value < 0.001, ****p value < 0.0001

Next, we investigated the effect of cisplatin and 17AAG as single drugs and in combination on apoptosis by applying the surface marker Annexin V, a marker for apoptosis. Cisplatin significantly increased apoptosis in RIVA, OCI-Ly7, and DB with 3.4-, 2.8-, and 1.9-fold higher Annexin V-positive cells, respectively, compared to the control (*p* = 1.28 × 10^–8^, *p* = 3.98 × 10^–6^, and *p* = 4.37 × 10^–7^, respectively) (Fig. 5A-C). Likewise, exposure to 17AAG induced apoptosis in RIVA and DB in comparison to the control (5- and 1.4-fold, *p* = 1.03 × 10^–9^ and *p* = 1.41 × 10^–5^, respectively) but not in OCI-Ly7 cells (Fig. 5A–C). Combination of 17AAG with cisplatin significantly increased apoptosis in DLBCL cells in most cases (Fig. 5A-C), supporting the synergistic effect observed for the combinatory treatment in the cell viability analyses. Specifically, when compared to cisplatin treatment, the combination increased apoptosis levels in RIVA and DB (*p* = 1.55 × 10^–6^ and *p* = 1.12 × 10^–2^, respectively), but not in OCI-Ly7, whereas the drug combination significantly increased the levels in all three cell lines compared to 17AAG-treated cells (RIVA (*p* = 5.40 × 10^–4^), OCI-Ly7 (*p* = 2.79 × 10^–4^) and in DB (*p* = 2.76 × 10^–7^)) (Fig. 5A–C).

### Combination of cisplatin and 17AAG activates the DNA damage response

Cisplatin induces DNA damage, resulting in activation of DNA repair mechanisms, and since Hsp90 has been shown to affect many DNA repair pathways, we examined whether the combination of cisplatin and 17AAG would lead to an increase in DNA damage, which could plausibly explain the observed synergy.

**Fig. 6:**
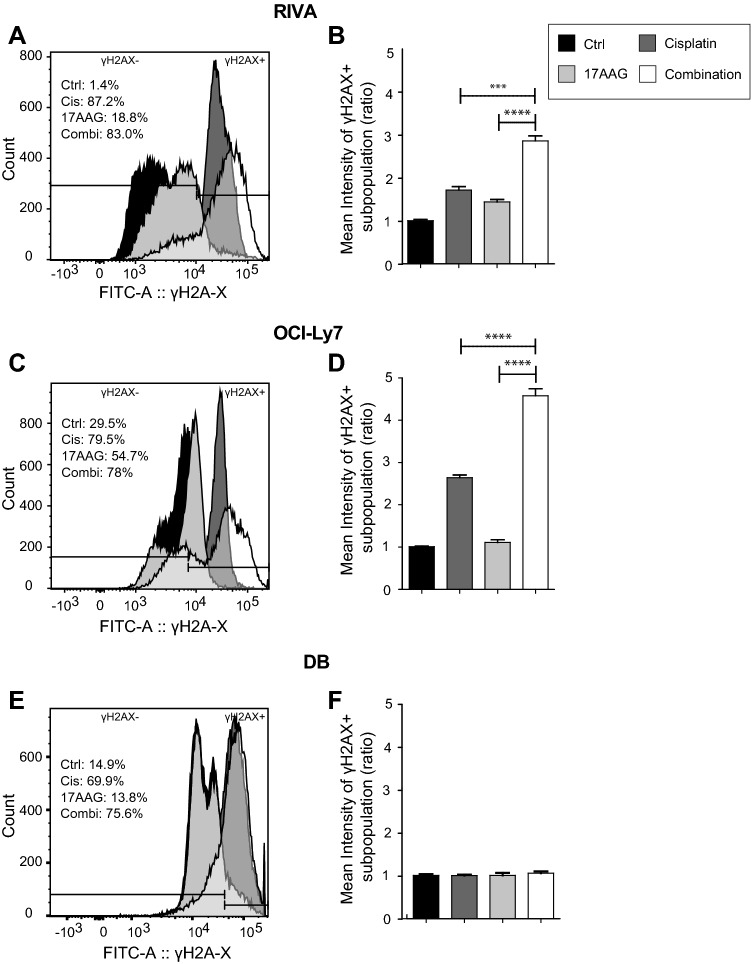
DNA damage response after treatment measured by anti-γ-H2AX staining. Three DLBCL cell lines were treated with vehicle, single drugs or combination for 48 h. **A** + **B** Results for RIVA. Figure 6A shows histograms for γH2AX for all treatments, where the gate for γH2AX positive (γH2AX +) cells were defined using unstained samples. Percentages for γH2AX + cells are calculated from triplicates and written within the figure. Figure 6B shows γH2AX mean intensity for cisplatin- and combination-treated cells calculated and shown as a ratio to vehicle-treated cells, where controls are set to 1. Same panel set-up is shown for OCI-Ly7 (**C** + **D**) and DB (**E** + **F**). Histograms show one representative sample, and mean intensities (± SD) are calculated from triplicates from one representative experiment from each cell line (*n* = 2). Student’s Independent *t*-test was used for statistical analysis, *p value < 0.05, **p value < 0.01, ***p value < 0.001, ****p value < 0.0001

In all experiments, unstained samples were included to set gates defining positive (γH2AX +) and negative (γH2AX-) populations, and vehicle-treated samples to assess changes in γH2AX levels after drug treatment. Cisplatin treatment significantly increased the percentage of γH2AX positive cells (Fig. 6A + C + E), with 87.2% ± 0.3 γH2AX positive RIVA cells compared to 1.4% ± 1.1 of the controls (*p* = 1.79 × 10^–10^), 79.5% ± 1.4 γH2AX positive OCI-Ly7 cells compared to 29.5% ± 0.8 of the controls (*p* = 6.69 × 10^–7^), and 69.9% ± 2.2 γH2AX positive DB cells compared to the control with 14.9% ± 1.2 positive cells (*p* = 2.93 × 10^–6^). The Hsp90 inhibitor 17AAG only induced DNA damage in RIVA (18.8% ± 1.0 γH2AX positive cells) and OCI-Ly7 (54.7% ± 1.1 γH2AX positive cells) (*p* = 7.49 × 10^–6^ and *p* = 4.74 × 10^–6^, respectively*)* (Fig. 6A–D).

The combination of the two drugs increased the percentage of γH2AX positive cells for all examined cell lines in comparison to vehicle-treated samples (Fig. 6A + C + E) (RIVA: 83.0% ± 0.6 γH2AX positive cells, *p* = 2.29 × 10^–9^; OCI-Ly7: 78.0% ± 3.2 γH2AX positive cells, *p* = 1.42 × 10^–5^; DB: 75.6% ± 0.1 γH2AX positive cells, *p* = 1.18 × 10^–7^). In comparison to single drug exposure, the combination of cisplatin and 17AAG increased the percentage of γH2AX positive cells in DB (Fig. 6E), whereas the population of γH2AX positive OCI-Ly7 and RIVA cells only differed between the combination and 17AAG (Fig. 6A + C).

The intensity of yH2AX positive cells represents the number of yH2AX foci per cell and the total degree of DNA damage response [21]. Consequently, mean intensities of γH2AX positive populations were plotted for each cell line (Fig. 6B + D + F), showing a significantly higher intensity of the γH2AX positive cells from the combination therapy compared to cisplatin and 17AAG treatment alone in RIVA (*p* = 1.56 × 10^–4^, *p* = 4.86 × 10^–5^) and OCI-Ly7 (*p* = 4.54 × 10^–5^, *p* = 4.42 × 10^–6^), but not in DB. These results indicate that the synergistic effect of 17AAG addition to cisplatin treatment is mediated by induced DNA damage (Fig. 6), resulting in increased levels of apoptosis (Fig. 5).

## Discussion

In this study, we present robust data documenting that the Hsp90 inhibitor 17AAG enhances the anti-neoplastic effect of the commonly used chemotherapeutic drug cisplatin in DLBCL cell lines. To encompass the entire drug interaction landscape, we measured the combination effects across 7 DLBCL cell lines using both the Bliss Independence Model [16] and the Combination Index [19] to ensure robust and reproducible results. The cell lines with lowest sensitivity to cisplatin treatment (RIVA, OCI-Ly7 and DB) were the ones exhibiting the strongest response to the drug combination, which is interesting from a clinical perspective where patients with cisplatin resistant tumors could benefit from inhibition of Hsp90. To evaluate the mechanism behind the observed synergy, these cell lines were selected for further studies.

Analysis of apoptosis assays documented induced apoptosis of the combination treatment in comparison to single drug exposure, suggesting a mechanism behind the synergistic drug interaction observed in the viability measurements. The most prominent apoptotic effect of the treatment combination was observed in RIVA, which in addition was particularly sensitive to 17AAG treatment. Further studies including molecular profiling are needed to address the cause for this, reserved for future work.

As cisplatin exposure induces DNA adducts, the DNA damaging effect of the drug combination was evaluated by the anti-γH2AX antibody, which stains γH2AX foci forming at sites of damaged DNA. We show that cisplatin in particular induces a strong DNA damage after 48-h treatment, as expected, in all 3 analyzed cell lines. Of interest, a significant induction of DNA damage response for the drug combination in comparison to cisplatin was observed in two of the three investigated DLBCL cell lines, suggesting that Hsp90 inhibition by 17AAG, leads to an impairment in DNA repair after DNA damage. 17AAG alone induced a modest DNA damage response compared to cisplatin but a significant effect compared to the treatment control in RIVA and OCI-Ly7. However, the lack of increased DNA damage for 17AAG alone and the combination treatment in DB could be explained by the most synergistic concentration being four times lower than in RIVA and OCI-Ly7. In agreement, the combination of cisplatin and an Hsp90 inhibitor has been shown to decrease cell viability and induce DNA damage response in ovarian and head and neck cancer cells [23–25].

In general, primary DLBCL biopsies display high levels of genomic instability and DNA damage [26, 27], which makes this tumor type heavily reliable on DNA repair and cell cycle check-point activation. This makes up the rationale for cisplatin treatment in DLBCL, where an additional hampering of the DNA repair machinery using Hsp90 inhibitors could be beneficial when attempting to improve clinical outcome and overcome treatment resistance, especially in the most resistant patients.

Previous studies have shown that deficiency of BRCA1, a client to Hsp90, sensitize cells to 17AAG treatment [28], and since tumor cells expressing high levels of BRCA1 are intrinsically resistant to irradiation and several chemotherapeutics [28, 29], Hsp90 inhibitors could be used in a therapeutic strategy in treatment regimens activating a DNA damage response. Another client of Hsp90 is the BRCA2 protein, which promotes RAD51-medieated DNA repair at DSB sites. 17AAG treatment causes increased BRCA2 degradation and thereby impairment of RAD51 and reduced activation of proteins involved in DNA repair. This has in fact already been shown when treating human lung adenocarcinoma cells with the Hsp90 inhibitor ganetespib [30]. In addition, a study in homologous repair (HR) proficient ovarian cancer cells showed that 17AAG suppressed HR DNA repair and enhanced carboplatin and olaparib responses [31]. Looking into other DNA repair pathways, several studies have suggested the mismatch repair pathway protein MutS homologue 2 (MSH2) as an interesting player in cisplatin response in cancer. A CRISPR screen in muscle invasive bladder cancer revealed MSH2 as the top candidate gene mediating cisplatin resistance [32], and low MSH2 expression has been linked to a longer survival in lung cancer patients treated with adjuvant cisplatin-based chemotherapy [33]. Additionally, 17AAG was shown to downregulate MSH2 in non-small cell lung carcinoma cells enhancing pemetrexed-induced cytotoxicity [34]. Studies focusing on the HR- and mismatch repair pathways in relation to 17AAG + cisplatin response in DLBCL are warranted to decipher the role of DNA repair and Hsp90 inhibitors in cisplatin and other DNA damaging drug responses in DLBCL. However, Hsp90 has numerous client proteins and, thus, its mechanisms of action could involve other systems than DNA repair pathways.

Many clinical studies have investigated Hsp90 inhibitors for their anti-cancer effect [35, 36], but currently no approved treatments are in clinical use [37]. Several large DLBCL patient cohorts exist including CHOP and/or R-CHOP response data and molecular profiling [38–40]; however, to date, there are no large-scale studies with comprehensive data on cisplatin-based therapy response and molecular profiling data available. Such clinical cohorts could greatly improve our understanding of the molecular groundwork for cisplatin response, setting the stage for individualized treatment strategies, and making it possible to test Hsp90, among other biomarkers, as a prognostic marker in relapsed DLBCL treated with cisplatin-based therapies.

In conclusion, we demonstrated that the Hsp90 inhibitor 17AAG exhibits a synergistic effect in DLBCL cells when treated in combination with cisplatin. The combination induced apoptosis and activated a stronger DNA damage response compared to single drugs alone. Our data support the implementation of Hsp90 inhibition in the treatment of relapsed DLBCL in combination with current cisplatin-based treatment regimens, to sensitize cisplatin insensitive cells to treatment.

## Supplementary Information

Below is the link to the electronic supplementary material.

Supplementary file1 (PDF 1623 KB)

## Data Availability

All data generated during this study are included in this article and supplementary information. Gene expression data can be accessed through the National Center for Biotechnology Information (NCBI) Gene Expression Omnibus (GEO).
