# Supplementary material for: Hsp90 inhibition sensitizes DLBCL cells to cisplatin
Source: Cancer Chemother Pharmacol. 2022 Feb 21;89(4):431–40. doi: 10.1007/s00280-022-04407-5 (PMC8956557; doi:10.1007/s00280-022-04407-5)
Supplement: Supplementary file 1 — Supplementary file1 (PDF 1623 KB) [file 280_2022_4407_MOESM1_ESM.pdf]

Title: Hsp90 inhibition sensitizes DLBCL cells to cisplatin
Journal: Cancer Chemotherapy and Pharmacology

Linnéa Schmidt[1,2], Issa Ismail Issa[1,2], Hulda Haraldsdóttir[1], Jonas Laugård Hald[1], Alexander Schmitz[1], Hanne Due[1], Karen Dybkær[1,2,3]
[1]Department of Hematology, Aalborg University Hospital, Aalborg, Denmark.
[2]Department of Clinical Medicine, Aalborg University, Aalborg, Denmark; and
[3]Clinical Cancer Research Center, Aalborg University Hospital, Aalborg, Denmark

Corresponding author:
Karen Dybkær, Department of Hematology, Aalborg University Hospital, Søndre Skovvej 15, 9000, Aalborg, Denmark.
k.dybkaer@rn.dk

Supplementary Materials

SUPPLEMENTARY FIGURE LEGENDS

Supplementary Figure 1. Overview of the drug combination experiments. To the left, a drug combination matrix with Bliss scores is shown for 17AAG and cisplatin in RIVA. On the y-axis doses of 17AAG can be seen (0.17-2.72 µg/mL) and on the x-axis doses of cisplatin are shown (0.42-6.8 µg/mL). Dark blue indicates a low Bliss score, thus a strong synergy. In total, 25 different combinations were tested in 7 different DLBCL cell lines. The right-side graph visually explains the data points seen in one horizontal line in the drug combination matrix as a dose-response curve (using ratio viability compared to vehicle-treated controls), i.e. a fixed 17AAG concentration and all 5 doses of cisplatin. The grey line indicates the viability ratio compared to vehicle-treated control for the fixed 17AAG dose (in this example 0.68 µg/mL17AAG), and the line is there only to ease interpretation.

Supplementary Figure 2. Drug combination raw ratio data and Bliss scores for all 7 DLBCL cell lines. Each horizontal panel are data for one DLBCL cell line. Left panels show raw ratio values (compared to vehicle-treated controls), dark red indicating a higher viability and white indicating a low viability. The corresponding Bliss score matrices are shown in the middle panel, dark blue indicating a low Bliss score, and right panels show graphs visually explaining the data points seen in one horizontal line in the drug combination matrix as a dose-response curve (using ratio viability compared to vehicle-treated controls), i.e. a fixed 17AAG concentration and all 5 doses of cisplatin. The grey line indicates the viability ratio compared to vehicle-treated control for the fixed 17AAG dose. Grey lines are only added to ease interpretation.

Supplementary figure 3. Correlation between 17AAG response and mRNA expression of *HSP90* genes. The area under dose-response curve (AUC) for 17AAG is displayed as a function of *HSP90* mRNA expression in the 7 DLBCL cell lines when untreated. Expression levels have been Log_2-normalized. Pearson correlation coefficients and p values have been calculated for each gene, in order to assess the level of correlation. *Hsp90AA1*: Heat shock protein 90 α A1, *Hsp90AB1*: Heat shock protein 90 α B1, *Hsp90B1*: Heat shock protein 90 β 1.

Supplementary Figure 4. Combination index for combination in 4 DLBCL cell lines. A combination index, an alternative estimation of combination effect, was calculated from the drug combination screen. On the top a table with Combination index (CI) for SU-DHL-4, HBL-1, SU-DHL-5 and NU-DHL-1 is shown. The three columns on the left show the single doses in five different combinations, and the four columns to the right show the CI for each cell line. ON the bottom, a visualization of the CI in the four DLBCL cell lines can be seen. A CI > 1 is an antagonistic interaction, a CI = 0 equals no, or additive, drug interaction, and a CI < 1 equals a synergistic drug interaction.

Supplementary Figure 1

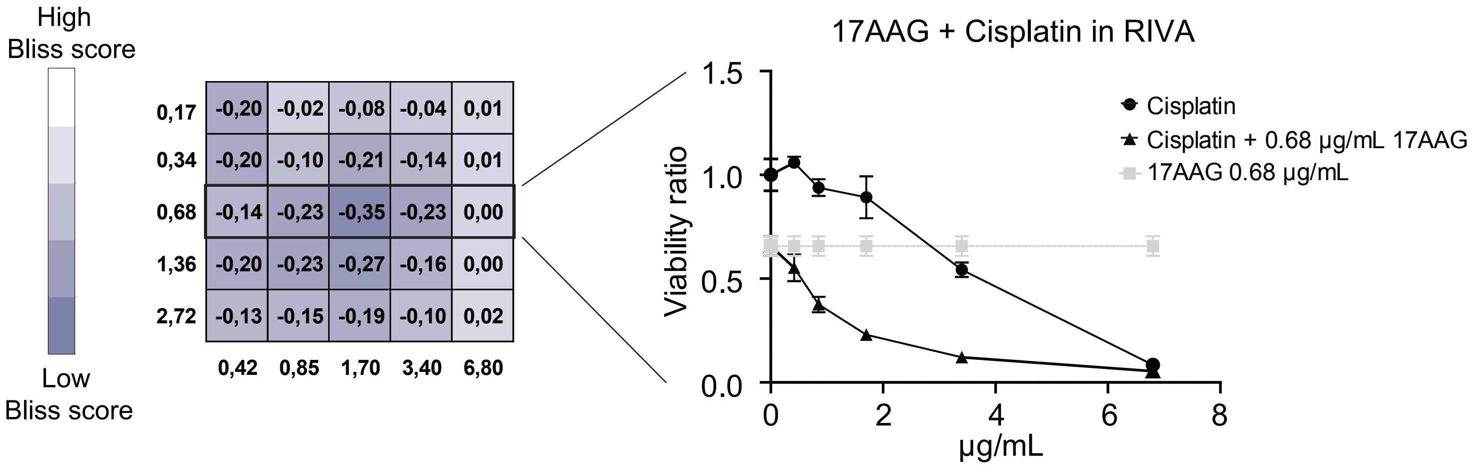

Supplementary Figure 2

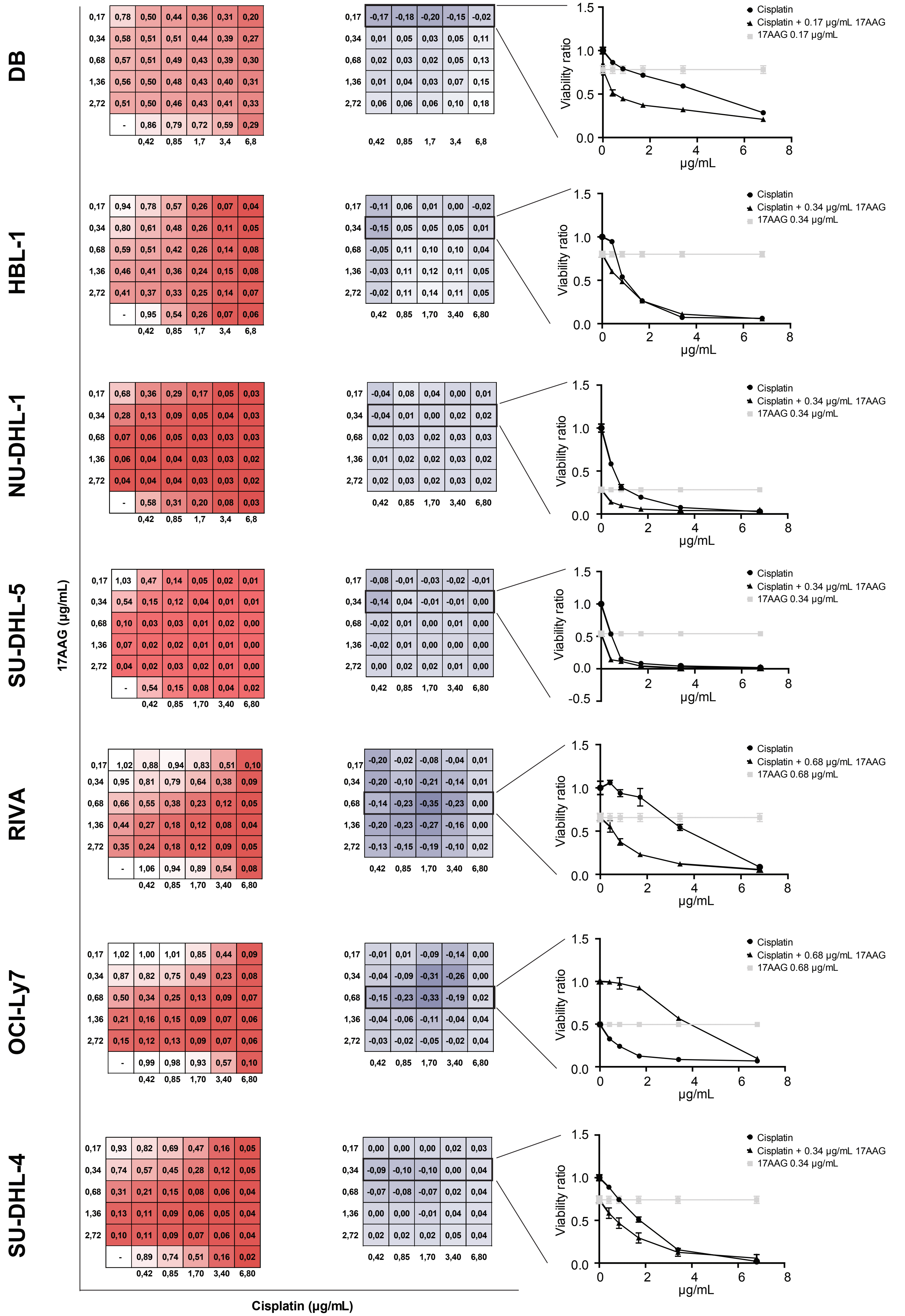

Supplementary Figure 3

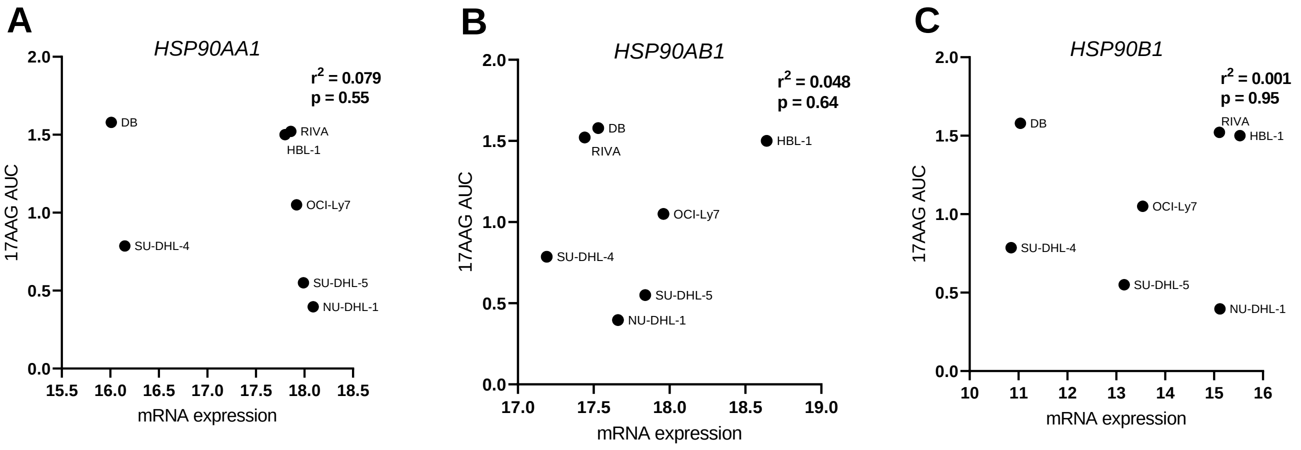

Supplementary Figure 4

Dose Cisplatin	Dose 17AAG	Combination number	CI SU-DHL-4	CI HBL-1	CI SU-DHL-5	CI NU-DHL-1
0.42	0.34	1	1.02	0.79	0.68	0.64
0.85	0.34	2	1.09	0.90	0.89	0.62
1.70	0.34	3	1.14	0.93	0.69	0.56
3.40	0.34	4	1.17	1.03	0.47	0.77
6.80	0.34	5	1.36	1.32	0.82	1.09

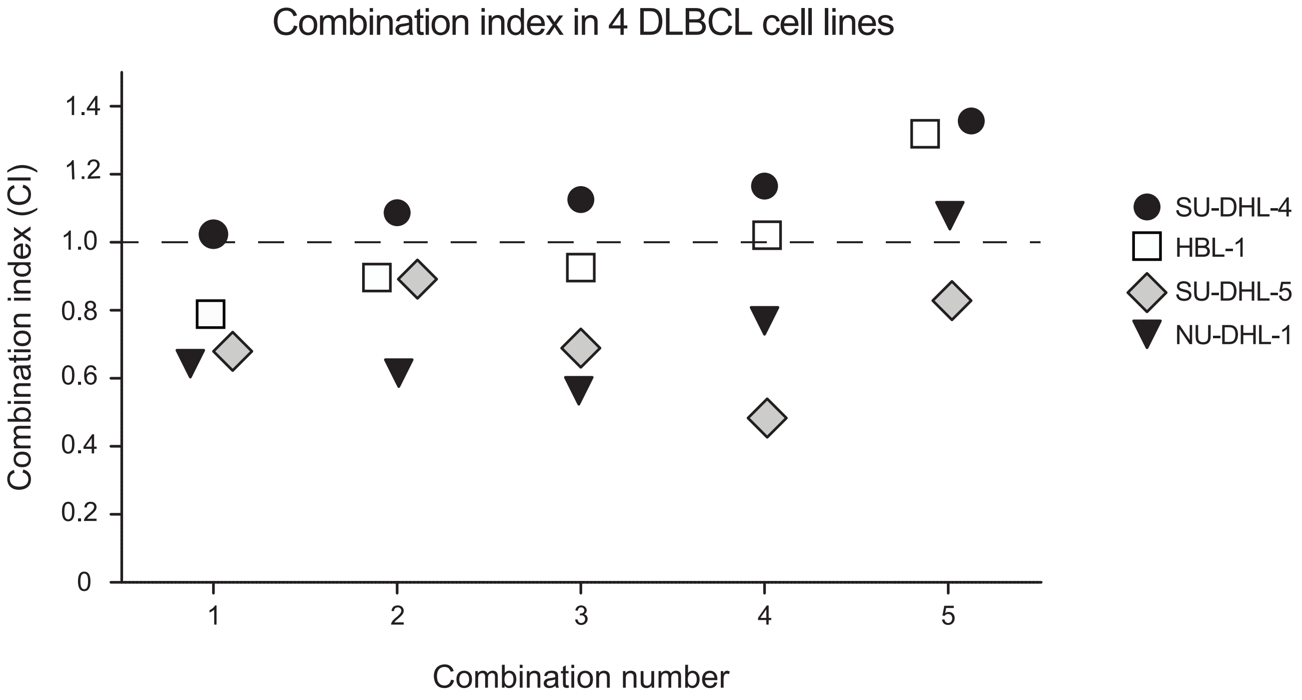

Table S1. Seeding concentrations for 7 DLBCL cell lines

	**Seeding concentrations***
DB	0.15 × 10^6 cells/mL
HBL-1	0.30 × 10^6 cells/mL
NU-DHL-1	0.25 × 10^6 cells/mL
SU-DHL-5	0.60 × 10^6 cells/mL
RIVA	0.25 × 10^6 cells/mL
OCI-Ly7	0.45 × 10^6 cells/mL
SU-DHL-4	0.30 × 10^6 cells/mL

* in 96-well plates

Table S2. IC50 for 7 DLBCL cell lines

	IC50 (µg/mL)	
	**Cisplatin**	**17AAG**
DB	3.59	2.24
HBL-1	1.22	1.39
NU-DHL-1	0.39	0.20
SU-DHL-5	0.35	0.53
RIVA	3.08	1.44
OCI-Ly7	3.56	0.92
SU-DHL-4	1.35	0.59
